# Male partner approval on the use of modern contraceptive methods: factors determining usage among couples in Kibaha district, Tanzania

**DOI:** 10.1186/s40834-020-00107-8

**Published:** 2020-04-17

**Authors:** Judith Msovela, Anna Tengia–Kessy, Susan F. Rumisha, Daudi O. Simba, David P. Urassa, Gernard Msamanga

**Affiliations:** 1 National Institute for Medical Research, Dar es Salaam, Tanzania; 2 Department of Community Health, Muhimbili University of Health and Allied Sciences, Dar es Salaam, Tanzania; 3 Malaria Atlas Project, Big Data Institute, Li Ka Shing Centre for Health Information and Discovery, University of Oxford, Oxford, UK; 4 Formally, Professor, Department of Community Health, Muhimbili University of Health and Allied Sciences, Dar es Salaam, Tanzania

**Keywords:** Men, Use, Modern contraceptive, Methods, Tanzania

## Abstract

**Background:**

Men in developing countries play an important role in the adoption of family planning (FP), either as actual users or supporters of their partners. Notwithstanding the universal knowledge on the contraceptive methods, their approval and use have been low among men in Tanzania. This study determined the magnitude and factors that influence men to use or approve the use of modern contraceptive methods with their spouses.

**Methods:**

A cross sectional, community-based study was conducted in Kibaha, Pwani region in 2014. A total of 365 randomly selected married and cohabiting men; aged 18 to 60 years who had at least a child below the age of 5 years were interviewed using a structured questionnaire. Descriptive statistics were performed and associations between status of men using modern FP with their partners and potential factors were tested using Chi-square and Fisher’s exact tests as appropriate. Logistic regression model was fitted to determine significant factors associated with male use of the methods with their partners.

**Results:**

About 60 % of men (59.7%) reported to use modern FP methods. In the bivariate analysis, education level (odds ratio (OR) = 2.6, CI = 1.4–4.8; *p* = 0.002); men knowledge on any contraceptive method (OR = 24.1, CI = 7.3–79.9; *p* < 0.001); awareness of a nearby FP clinic (OR = 6.2, CI = 3.1–12.3; *p* < 0.001); number of children (OR = 2, CI = 1.1–3.6; *p* < 0.025) and presence of a provider during clinic visit (OR = 12.0, CI = 2.26–63.7; *p* < 0.004) were significantly associated with the use of FP. However, in the multivariable analysis, only knowledge on FP methods (adjusted odds ratios (AOR) =26.4; CI = 7.9–88.4, *p* < 0.001) and number of children a man had (AOR = 1.9; CI = 1.0–3.6, *p* = 0.039) remained significantly associated with the use of modern FP methods.

**Conclusion:**

This study has shown that for men to use family planning methods with their partners, knowledge of FP methods and number of children are critical factors. Visiting a FP center alone or with a spouse, and availability of FP provider (during visit) also influence this practice. These findings emphasize a need to increase knowledge on contraception and family planning services access among men.

## Background

Despite the available efforts, family planning (FP) use in Tanzania remains low. Data from the National Bureau of Statistics (NBS) show that only 29% of all women and 34% of married women in Tanzania use any contraceptive methods [1]. Tanzania is among ten countries with the highest number of maternal and neonatal deaths [2]. The current maternal mortality ratio is 556 deaths per 100,000 live births and neonatal mortality rate is estimated at 25 per 1000 live births [3]. It is reported that maternal mortality rates can significantly be reduced if contraceptive prevalence rate increases [4]. Since unplanned pregnancies and short birth intervals are highly associated with adverse outcomes in maternal, newborn and child health, use of FP could mitigate them. For example infant and neonatal mortality rates associated with AIDS could be averted by preventing unintended pregnancies and hence mother-to-child transmission of HIV [5, 6].

Available reports show various factors that contribute to low contraceptive prevalence, including men’s opposition to or noninvolvement in FP services despite their high level of awareness of the methods and their benefits [7, 8]. Studies in developing countries show that decisions and practice of FP are determined by the level of male support and involvement [9, 10]. Research findings further indicate that despite good attitude and self-acceptability of contraceptive use, the uptake of the methods in majority of women in Africa is influenced by men [11]. For example, 90% of women in Ghana reported limited access to FP services due to their husbands’ opposition [12]. Likewise, 65.8% of women in central Tanzania would use modern FP methods if their husbands support them [13]. Some studies show that women may opt to use FP methods secretly due to opposition to practice FP with their partners [14, 15].

Studies in FP services have mostly focused on women’s perspectives. For example, status of couple’s use of modern FP methods, determinants of use of the methods as well as awareness on the methods among men have been, in most cases determined by asking women. Different factors, than those mentioned by women, might be responsible for males’ acceptance on practicing modern FP with their partners. There is an information gap on the extent to which men use or support the use of modern FP methods with their spouses and also what factors influence their decisions. Therefore, the research information will inform programs in developing strategies to effectively engage men in using and or supporting the use of FP methods with their partners.

## Materials and methods

### Study site

The study was conducted in Kibaha district, Pwani region. The district comprises of 11wards, whereas the later are administrative structures for one single town or portion of a bigger town which are further subdivided into streets and villages for urban and rural wards respectively. The district has a population of 75,899 of which adult male are 15,598 and women of reproductive age (15–49 years) are 19,015. There are 24 health facilities of which 16 are public and the only facilities providing FP services.

### Study design, sample size and sample selection

This was a quantitative cross sectional, community-based study conducted in Kibaha district in 2014. A multistage stratified sampling technique was used to select respondents. The eleven wards were stratified into urban and rural. From each stratum, one ward was randomly selected whereby Mlandizi (population 17,318; NBS, 2013) and Soga (population 4713; NBS, 2013) were selected to represent urban and rural strata respectively. Three streets (Mlandizi Kati, Kibwende and Msufini) were randomly selected out of the 10 streets of Mlandizi ward. Similarly, two villages (Soga and Vikuge) were selected from Soga ward which has five villages. From each selected street/village, the list of eligible men was prepared with the assistance of the village/street administrations. Inclusion criteria were men aged between 18 to 60 years, with at least a child under the age of 5 years and living with a spouse or partner during the survey. Exclusion criteria included men whose partners were beyond reproductive age i.e. aged above 49 years.

The minimum sample size for the study was obtained using the formula for calculating sample sizes for cross-sectional surveys [16], with the assumption that a third (38%) of couples in the region use contraceptives together [3]. The margin of error was set at 5% and the non-response rate was also 5%. These parameters provided a minimum sample of 376, of which were randomly selected from the prepared lists. The sample was split between the two wards using weights calculated based on the population size, and then taken proportionally based on the eligible men within the villages/streets.

### Data collection procedures and analysis

An interview schedule with both open and close-ended questions was used to collect data. The questionnaire was administered to the selected men at their homes except for few who decided to be interviewed at their working places since it was unlikely to get them at home during the day time. Interviews were conducted by the principal investigator and three research assistants with experience in data collection. The research assistants were oriented on the study objectives, the research tool and ethical aspects relevant for the study before embarking on data collection. As part of quality assurance, the process of data collection was supervised by regular follow-ups and discussions with the research assistants to address any problems encountered. Filled-in questionnaires were checked for completeness and accuracy of the collected data and rectifications made with the respective research assistant as appropriate. Responses from open-ended questions were categorized according to their similarities and then coded. Data were double entered by two independent data entrants in EpiData version 3.1 software. The data were cleaned and then exported to STATA version 11 (Stata Corp., College Station, TX) for analysis.

Analyses were performed for all study respondents and for a subset of those who ever visited FP clinic, either alone or with a partner. Descriptive statistics were done and are presented in the form of frequencies and proportions. Statistical analysis for associations between the dependent variable with each potential determinant was done using Chi-square test and Fisher’s exact test as appropriate. The outcome variable for this study was men use of FP methods with their partners (a binary variable: 1 = use, 0 = not use). Independent variables included demographic characteristics of the men such as age; marital status and education level; and health system factors such as distance to health facility and availability of service providers. To establish factors associated with use of FP, classical logistic regression model was fitted. Factors with *p*-value < 0.20 in the binary analysis were considered for a multivariable analysis. Odds ratios and their 95% confidence intervals are presented to quantify the association. Significance was considered at *p*-value < 0.05. Hypothesizing that, male attendance to the FP centre, alone or with their partner might influence the usage, we analyzed a subset of those men who ever visited FP centre and treated them as a separate population. In some cases, results of the two sets; the all men and those visited FP centre are compared and discussed.

## Results

### Sociodemographic characteristics of respondents

A total of 365 men (response rate was 97.1%) were interviewed; 239 (65.4%) from urban (Mlandizi) and 126 (34.6%) from Soga (rural) wards (Table 1). Their mean age of the respondents was 35 years and the highest proportion (43.8%) was in the age group of 28–37 years. About three-quarters of the men (72.3%) reported to be married and a quarter had either one or two children. Whereas 239 of them (65.5%) had completed primary education, only a tenth had completed secondary education. The highest proportion (41.1%) were practicing agriculture as an economic activity.

**Table 1:** Background information of the respondents

Variables (***N*** = 365)	Frequency (Percent)
**Residency**	
Urban	239 (65.4)
Rural	126 (34.6)
**Age in years**	
18–27	71 (19.4)
28–37	160 (43.8)
38–47	90 (24.7)
> 47	44 (12.0)
**Education level**	
No education	31 (8.5)
Primary	260 (71.2)
Secondary and more	74 (20.3)
**Number of children:**	
One	98 (26.8)
Two	89 (24.3)
Three	73 (20.0)
More than three	105 (28.9)

### Use of modern FP by sociodemographic characteristics

According to self-reports, about 60% (218/365) of all respondents were using any FP methods with their spouses at the time of the survey, commonly the male condom (24.6%) followed by injectable contraceptives (22%) and oral pills 20.6%. Other methods used include, implant (14.7%); calendar (12.8%); and 5.3% used IUD (6), withdraw (3), BTL (2) and eleven abstained. Some of the respondents reported using dual methods such as condom and pills; and condom and calendar. Men who were not using FP methods had various reasons including lack of knowledge about FP (62.3%) and fear of side effects (15.7%).

Despite Soga ward (rural) having 8 % more men who used FP with their partners compared to Mlandizi Kati (65.1% for Soga vs. 56.9% for Mlandizi Kati), the difference was not statistically significant. Proportionally, men who reported to be formally married were more likely to use FP with their partners (62.1%) than their counterparts (53.5%) who were cohabiting. Generally, men who had completed primary education were significantly more likely to use of FP methods with their partners compared to their counterparts who had no education (*p* < 0.05). On the other hand, age did not significantly influence men to use FP methods with their partners. Men who had a single child were less likely to report use of FP method compared to those who had two or more children (p < 0.05). Furthermore, FP use was more in a polygamous marital union (Table 2).

**Table 2:** Background characteristics of study population categorized as all vs. those visited FP Centre and their association with usage of FP with partner

All males (***n*** = 365)				***p***-value	Visited FP Centre (***n*** = 55)		All, N(%)	***p***-value
Characteristics	Use, N(%)	No use, N(%)	All, N(%)		Use, N(%)	No use, N(%)		
**Education**								
No education	22 (10.1)	30 (20.4)	52 (14.3)	< 0.05	2 (5.1)	3 (18.8)	5 (9.1)	> 0.05
Primary	157 (72)	82 (55.8)	239 (65.5)		24 (61.5)	8 (50)	32 (58.2)	
Secondary +	39 (17.9)	35 (23.8)	74 (20.3)		13 (33.3)	5 (31.3)	18 (32.7)	
**Age (in years)**								
18–27	38 (17.4)	33 (22.5)	71 (19.5)		11 (28.2)	3 (18.8)	14 (25.5)	< 0.01
28–37	94 (43.1)	66 (44.9)	160 (43.8)	> 0.05	15 (38.5)	5 (31.3)	20 (36.4)	
38–47	56 (25.7)	34 (23.1)	90 (24.7)		9 (23.1)	3 (18.8)	12 (21.8)	
> 47	30 (13.8)	14 (9.5)	44 (12.1)		4 (10.3)	5 (31.3)	9 (16.4)	
**Residency**								
Rural	82 (37.6)	44 (29.9)	126 (34.5)	> 0.05	15 (38.5)	4 (25)	19 (34.6)	> 0.05
Urban	136 (62.4)	103 (70.1)	239 (65.5)		24 (61.5)	12 (75)	36 (65.5)	
**Type of marital Union**								
Single	210 (96.3)	136 (92.5)	346 (94.8)	0.05	37 (94.9)	14 (87.5)	51 (92.7)	< 0.001
Polygamous	8 (3.7)	11 (7.5)	19 (5.2)		2 (5.1)	2 (12.5)	4 (7.3)	
**Number of Children**								
One	49 (22.5)	49 (33.3)	98 (26.9)	< 0.05	10 (25.6)	6 (37.5)	16 (29.1)	> 0.05
Two	59 (27.1)	30 (20.4)	89 (24.4)		12 (30.8)	1 (6.3)	13 (23.6)	
Three	46 (21.1)	27 (18.4)	73 (20)		7 (18)	5 (31.3)	12 (21.8)	
More than three	64 (29.4)	41 (27.9)	105 (28.8)		10 (25.6)	4 (25)	14 (25.5)	
**Total**	**218**	**147**	**365**		**39**	**16**	**55**	

### Use of FP methods and access to reproductive health services

In spite of only 55 (15%; 55/365) men reporting to have ever accessed a FP service point for contraceptive methods, 74.5% (234/314) of them could reach a nearby service centre by walking for less than 30 min. Over 70 % (70.9%, 39/55) of these men reported to use FP methods with their spouses. There was a significant difference (*p* < 0.001) in proportion of users between men who visited health facility (HF) for FP services (70.9%) and those who did not visit (57.7%, 179/310). In addition, the usage was significantly lower in the overall men population in the study (59.7%) compared to the subset of those who visited FP centres (*p* < 0.001). This was more profound with regards to age and type of marital union. More young men (18-27 yrs) who visited these centres were using FP methods with partners (17.4% in all vs. 28.2% in those visited); and, similarly for men in polygamous marital union (3.7% in all vs. 5.1% in those visited). There was a slight difference in number of children among users and none users in the overall men population. Zooming in at the subset of those who visited the HFs for FP, it is noted that there is a significantly higher proportion (31.3%) of older men (> 47 years) who didn’t use FP with partners as compared to when the full set was analyzed which was 9.5% (*p* < 0.01). Level of education was significantly associated with usage in the full set, although this was not the case when the subset of those visiting centres was analyzed (Table 2).

Knowledge on FP and cost for visiting health facility was associated with the use of FP. Respondents who had knowledge on FP methods were more likely to use the methods with their partners as compared to their counterparts who had no knowledge on the subject (Table 3). This was strongly indicated in all men (*p* < 0.001) but weakly for those who visited the FP centres (*p* < 0.05). Knowledge of a nearby FP center was good in these men but more for those who used FP methods. Moreover, travelling cost to reach FP centre had a slight effect on the use of FP. In both sets of analysis, men who did not use FP claimed to incur more cost to reach the centers than those who used the methods (*p* < 0.05).

**Table 3:** Knowledge and Health System Factors associated with FP usage with partner in males categorized as all vs. those visited FP Centre

Factor	All males	No use	Total	***p***-value	Visited FP Centre		Total	***p***-value
	Use				Use	No use		
**Knowledge of FP Method (any), N (%)**								
Yes	215 (98.6)	110 (74.8)	325 (89)	< 0.001	39 (100)	15 (93.8)	54 (98.2)	< 0.05
No	3 (1.4)	37 (25.2)	40 (11)		0 (0)	1 (6.3)	1 (1.8)	
**Knowledge of nearby FP Centre, N (%)**								
Yes	206 (94.5)	108 (73.5)	314 (86)	< 0.001	37 (94.9)	16 (100)	53 (96.4)	< 0.05
No	12 (5.5)	39 (26.5)	51 (14)		2 (5.1)	0 (0)	2 (3.6)	
**Average time to reach nearby FP Centre**								
Time in minutes (SD)	14.4 (15.8)	14.9 (26.4)	14.7 (23.2)	> 0.05	17.6 (22.2)	15.9 (17.9)	17.1 (20.9)	> 0.05
**Average cost for a return trip to the FP centre**								
Cost in TZS (SD)	1870 (1390)	2533 (2838)	2118 (1989)	< 0.05	2000	8000	5000 (4242)	< 0.05

Results of the bivariate logistic regression model indicated that men’s knowledge on any FP method, knowledge on location of a nearby FP centre and number of children to be associated with men use of FP methods (Table 4). The only background characteristic that remained significantly associated with men use of FP methods with their spouses was education level (*p* = 0.002). In health system factors, finding a family planning provider at the time of visit was significantly associated with the use of FP methods.

**Table 4:** Bivariate analysis to determine factors associated with men’s use of family planning with their spouses

Variable	OR	95% CI	***p***-value
Ward (ref = Mlandizi)			
Soga	1.4	(0.9;2.2)	0.131
Village (ref = Kibwende)			
Mlandizi K	1.7	(0.8;3.3)	0.136
Msufini	0.5	(0.2;1)	0.066
Soga	1.6	(0.8;3.5)	0.195
Vikuge	1.5	(0.6;3.3)	0.36
Marital status (ref = married)			
Cohabiting	0.7	(0.4;1.1)	0.132
Type of marital union (ref Monogamous)			
Polygamous	0.5	(0.2;1.2)	0.115
Education level (ref = no education)			
Primary	2.6	(1.4;4.8)	0.002^*^
Secondary and above	1.5	(0.7;3.1)	0.251
Knowledge on any contraceptive method	24.1	(7.3;79.9)	< 0.001^*^
Knowledge of nearby FP centre	6.2	(3.1;12.3)	< 0.001^*^
Living in urban area	0.7	(0.5;1.1)	0.131
Age categories (in years) ref.: 17-27yrs			
28–37	1.2	(0.7;2.2)	0.459
38–47	1.4	(0.8;2.7)	0.267
48+	1.9	(0.8;4.1)	0.122
Number of children (ref: One)			
Two	2	(1.1;3.6)	0.025^*^
Three	1.7	(0.9;3.2)	0.091
More than three	1.6	(0.9;2.7)	0.117
Distance to the nearest FP centre	1.0	(0.99;1.01)	0.839
Availability of FP provider (during visit)	12.0	(2.26;63.7)	0.004^*^

^*^P value < 0.05

Variables such as residency; marital status; type of marital union; level of education; knowledge of contraceptive methods and availability of a nearby family planning centre; number of children and; availability of provider during visit to delivery point were qualified and fitted in multivariable analysis model. The results reveal that men with knowledge on any FP methods stand a 26 times higher chance of using them compared to men who were not knowledgeable. Furthermore, men who had more than one child had twice the chance of using FP methods than those with one child (Table 5).

**Table 5:** Multivariable analysis on the factors associated with men’s use of FP with spouse

Variable	OR	95% CI	***p***-value
Knowledge on any contraceptive method	26.4	(7.9;88.4)	< 0.001
Number of children (ref: One)			
Two	1.9	(1;3.6)	0.039
Three	2	(1;3.9)	0.042
More than three	2	(1.1;3.8)	0.022

## Discussion

This study aimed at establishing the extent to which men in Pwani region use FP methods with their partners and the determinants. This is among few research works that have attempted to explore these factors from the male perspectives. Almost two-thirds of men reported using modern FP methods with their partners in Kibaha. This proportion is higher than what has been observed in a study involving women (35%) in central Tanzania [13]. Men respondents in Kibaha might have exaggerated the actual situation of using FP methods with their partners as reflected in the reported low contraceptive prevalence (38.3%) among married women in Pwani region [1]. However, some studies have also reported higher proportions of men using FP methods, 77.5% in Ethiopia [17] and 89% in Nigeria [8]. This difference could be attributed to the, differences in study settings in terms of exposure to various interventions and social cultural issues.

Men use of FP was found to be associated with several factors. Level of education has shown to influence use of FP methods, whereby respondents who had completed primary education used FP methods more than those who had no education. Similar findings have been reported by a study in Nigeria which showed that men with higher level of education were more likely to use the methods than their counterparts with less schooling [18].

Majority of men had knowledge on FP in terms of awareness of various contraceptive methods and where the commodities could be accessed, and this positively influenced on men using FP with their partners. This finding is similar to other studies in the low income countries which show that men who had knowledge of the source of FP commodities were significantly more likely to use contraception [19]. Likewise, a study in Ghana reported that lack of adequate knowledge contributes to low use of contraceptives among men [20]. These findings might suggest a need to increase promotional efforts in order to increase awareness on various methods and their availability.

Number of children was positively associated with male use of FP methods with their partners. This finding conforms to a previous study among sexually active men, which show that men were more likely to use contraceptive methods if they had at least three children [21].

Men visit to health facilities, either as clients or accompanying their partners provides an opportunity for couple counseling which enhance joint decision making on using the methods. This study has revealed that very few men visited health facilities for FP services. Similarly, other studies in resource limited countries reported low proportions of men who visit FP centres [22]. Historically, FP services have been female-oriented and hence perceived as women concerns [7], a phenomenon which might have put men away. Another reason for men not accessing FP service centres could be their perception that visiting such centres are a waste of time [23]. In the current study, distance and associated cost incurred could be the reasons for fewer men to visit health facilities and so use of modern family planning services. Due to limited resources, couples may decide for only one of them to visit the service centre when in need of such services, and in most cases, this is the woman. Interestingly, a clear difference in using FP methods with their partners was observed between men who attended FP centers and those who did not attend. This finding underscores the need to increase efforts to address missed opportunities for men who do not visit reproductive health clinics.

Finding a provider when visiting a health facility for FP services, has shown to influence men use of the methods with their partners. Other studies in Tanzania and Uganda have reported similar results that unavailability or inaccessible health providers as barriers to men in accessing reproductive health services [24, 25]. Finding a provider might have a positive influence as men may get the opportunity of being counseled and appropriately educated to make informed choices.

### Study limitations

This study included married or cohabiting men but other sexually active men were not represented. Understanding of family planning knowledge and practice of young unmarried men is important as this category of men could provide an entry point for education which will improve their sexual and reproductive health in premarital and marital life. Moreover, as this was a cross-sectional study, it is difficult to make a causal inference. Further, this study was done only in Pwani region of which features and characteristics of men might not be representative with regard to culture, economics, ethnicity, women values and strength in decisions differences, hence interpretation should not be extrapolated directly to other settings.

## Conclusion

This study has shown that knowledge of FP methods among men and the number of children they have, play a significant role on the use of FP methods with their partners. Visiting a FP center alone or with a spouse also increases the chance for using contraception. Health delivery factors such as availability of FP service provider at the time of visit influence men use of modern family planning methods. These results call for more efforts to provide awareness to men on FP. Increasing implementation of existing effective interventions which aim at increasing awareness on FP among men at community and service delivery level is important for enhancing male partner involvement in modern contraception acceptance and use.

## Data Availability

The dataset used in this study are available from the corresponding author on reasonable request.

## Abbreviations

FP: Family Planning
OR: Odds Ratio
AOR: Adjusted Odds Ratio
NBS: National Bureau of Statistics
TDHS-MIS: Tanzania Demographic Health Survey- Malaria Indicator Survey
